# Measurement of relative transition strengths of ^133^Cs Rydberg D states using electromagnetically induced transparency

**DOI:** 10.1038/s41598-024-58385-0

**Published:** 2024-04-02

**Authors:** Shanxia Bao, Hao Zhang, Linjie Zhang, Liantuan Xiao, Suotang Jia

**Affiliations:** 1 Institute of Theoretical Physics, Shanxi Datong University, Datong, 037009 Shanxi China; 2 State Key Laboratory of Quantum Optics and Quantum Optics Devices, Institute of Laser Spectroscopy, Shanxi University, Taiyuan, 030006 Shanxi China; 3 Collaborative Innovation Center of Extreme Optics, Shanxi University, Taiyuan, 030006 Shanxi China

**Keywords:** Transition strengths measurement, Rydberg atoms, Electromagnetically induced transparency, Physics, Atomic and molecular physics, Atomic and molecular interactions with photons

## Abstract

Transition strengths between states are fundamental physical properties of atomic spectra. The differences in fine structure splitting of certain states are mainly attributed to the angular momentum parts of transition dipole matrix elements. These can be calculated by integrating the wave-functions theoretically and can be accessed by selecting corresponding polarizations of the exciting lasers experimentally. We measured the transition strengths ratios of *n*D$_{5/2}$/*n*D$_{3/2}$ via Rydberg electromagnetically induced transparency (EIT) by changing the powers and polarizations of probing and coupling lasers in a room temperature cesium vapor cell. The variation of the ratios on the principal quantum number *n* which ranges from 40 to 62 is also investigated. Theoretical and experimental results agreed with each other.

## Introduction

Rydberg atoms with principal quantum number *n* $$\gg $$ 1 possess exaggerated properties especially very long lifetime compared to low-lying states and very small transition dipole moment with respect to ground state^1^, which are attractive in quantum information or nonlinear optics^2–5^, also in field metrology^6,7^. Recently, electromagnetically induced transparency (EIT) has been extensively investigated in ladder-type system of Rydberg atoms^8–12^, and the linewidth of EIT spectrum is smaller than the broadening of lower states^13^. Then the quantum interference observed between dressed states in EIT^14^ has provided an effective tool for optical sub-doppler detection of Rydberg states even in thermal vapor cell^15^. Relative transition strengths of different Rydberg states respect to ground state are significant parameters for measurement of fine-structure coupling constants and spectroscopic calibrations, as well as in applications such as Rydberg-atom-based metrology^16^. Especially for D state Rydberg atoms, whose fine structure splittings are more complex. Therefore a detailed study of the relative transition intensity of *n*D$$_{5/2}$$ and *n*D$$_{3/2}$$ is very meaningful for precise measurement based on Rydberg atoms. In early years, Cesium oscillator strengths are calculated using one-electron wave functions^17^ and Rydberg states of alkali atom’s oscillator strengths have been measured recently^18^. Intensity ratios of doublets arising from $$n 'S \rightarrow n $$ D$$_{5/2}$$,$$_{3/2}$$ transition have been measured in the two-photon absorption spectrum of rubidium (*n* = 5–9, *n*’ = 5) and cesium (*n* = 7–13, *n*’ = 6)^19^. Relative oscillator strengths of the diffuse and the sharp series of RbI have been measured in absorption and emission spectra^20^. Effective lifetimes of Rydberg states and observations of electric quadrupole transitions to Rydberg *n*D states are carried out in ultracold atoms^21–23^. The oscillator strengths (6P$$_{3/2}\rightarrow n $$S$$n $$D) and oscillator strengths ratios $$\eta $$($$n $$)=($$f _{D5/2}$$ )/($$f _{D3/2}$$) and $$\rho $$($$n $$) = ($$f _{S1/2}$$($$n $$ + 2))/($$f _{D3/2}$$($$n $$)) have been measured by detecting ions signals of field ionization of cesium Rydberg atoms^24^. The ratio of D-line transition strengths have been measured in a thermal cesium vapor cell^25^.

In our experiments, we report the measurement of relative transition strengths ratios of fine structures of Rydberg states comprehensively, by varying combinations of polarizations and powers of probe and coupling beams, as well as different principal quantum number *n*. The strengths ratio *n*D$_{5/2}$/*n*D$_{3/2}$ are obtained by EIT spectroscopy and results are agreed with theoretical calculations.

## Experimental setup

**Figure 1:**
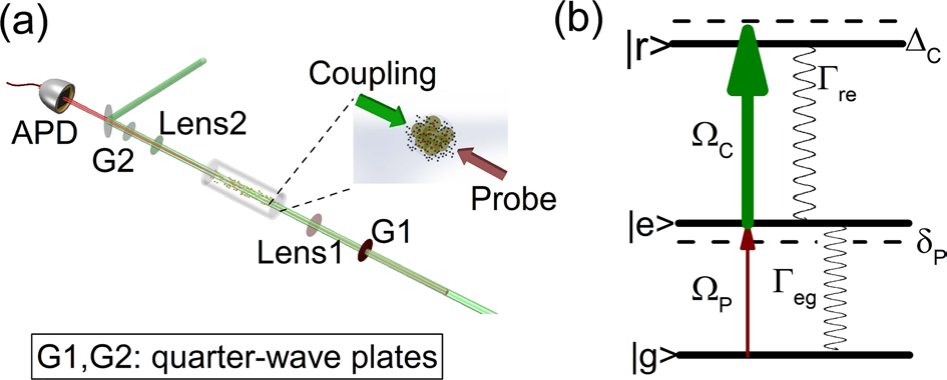
(**a**) Sketch of the experimental setup. The 852 nm probe and 510 nm coupling beams are counter-propagated and focused with lens1 and lens2 respectively to be 80 and 100 $\upmu$m in waist in the center of cell, and the G1/2 are quarter-wave plates to change the polarizations of two lasers. The frequency of probe laser is locked while that of coupling laser is scanned to avoid the doppler background. The probe beam is detected by a fast photodetector. (**b**) Energy levels of the ladder system, in which $\Omega _{P}$ and $\Omega _{C}$ are Rabi frequencies of probe and coupling lasers. $\Gamma _{eg}$ and $\Gamma _{re}$ are natural decay rates. $\Delta _{C}$ and $\delta _{P}$ are detunings of coupling and probing lasers with respect to the corresponding transitions.

The measurements are carried out in a cylindrical quartz cell with length of 4 cm and diameter of 2 cm of bottom filled with $$^{133}$$Cs atomic vapor of which the pressure is above saturation value as shown in Fig. 1a. The Rydberg states are obtained by two-photon excitation method, the dipole allowed transition is 6S$$_{1/2}$$(F = 4) $$\rightarrow $$ 6P$$_{3/2}$$(F’ = 5) $$\rightarrow $$ $$n $$D$$_{5/2,3/2}$$, and the corresponding energy scheme is shown in Fig. 1b. The probe and coupling beams are counter-propagated and focused to waist of 80 and 100 $$\upmu $$m respectively with two aspheric lens. The probe beam which drives the transition of 6S$$_{1/2}$$ (F = 4)$$\rightarrow $$6P$$_{3/2}$$ (F’ = 5) is provided by an external-cavity diode laser (DL100, Toptica) with the wavelength of 852 nm. While the coupling beam driving the transition of 6P$$_{3/2}$$ $$\rightarrow n $$D$$_{5/2,3/2}$$ is generated by a frequency-doubled laser system (TA-SHG pro, Toptica) with the wavelength of 510 nm. In order to get rid of the large doppler background, the frequency of probe laser is locked to a High-Finesse Fabry-Perot cavity constructed with Ultra-Low thermal Expansion (ULE) glass (ATF-6010-4, Stable laser system). The linewidth is smaller than 10kHz which provides a sufficient spectral resolution. Then an acoustic-optical modulator (AOM) is used in double-pass configuration to shift the frequency of probe laser to the resonance of 6S$$_{1/2}$$ (F = 4)$$\rightarrow $$6P$$_{3/2}$$ (F’ = 5) hyperfine transition. When the frequency of coupling beam is scanned through another AOM and is monitored and recorded by a wavelength meter (High Finesse-Angstrom WSU-30), the transmission of probe laser is detected with a fast photodetector (PDA36A-EC, Thorlabs). Then EIT spectra with flat background are observed and recorded by oscilloscope with bandwidth of 2 GHz which guarantees the accuracy of the experiment results.

## Theoretical description

In this paper, as mentioned above we performed Rydberg excitation of cesium atoms via intermediate state. The wavelengths of two lasers are large compared to the size of atoms, so it is sufficient to treat the system using the semi-classical method. Then the two light field can be written as E$_{i}$(t) = E$_{i,0}$cos(e$^{i\omega _{i}t}$+e$^{-i\omega _{i}t}$) where *i* = 1,2 for the first and second transition. The ground, intermediate and Rydberg states are denoted with$|g\rangle$, $|e\rangle$ and $|r\rangle$, respectively. The density matrix of the system is therefore

1 $$\begin{aligned} \rho =\left( \begin{array}{ccc} \rho _{gg}&{}\rho _{ge}&{}\rho _{gr}\\ \rho _{eg}&{}\rho _{ee}&{}\rho _{er}\\ \rho _{rg}&{}\rho _{re}&{}\rho _{rr}\\ \end{array}\right) \end{aligned}$$

The Hamiltonian of the 3-level ladder-type system consists of unperturbed $H_{0}$ and interaction between atoms and lights $H_{AL}$ as:

2 $$\begin{aligned} H=H_{0}+H_{AL} \end{aligned}$$

The transition dipole matrix elements of first and second excitation are denoted with $d _{eg}$ and $d _{er}$, while $E_{1,0}$ and $E_{2,0}$ represent the amplitudes of electric components of two lasers. Then we can define the Rabi frequencies describing the couplings between atoms and lights as $\Omega _{P}=\Omega _{eg}=\frac{-d _{eg}|E _{1,0}|}{h }$ and $\Omega _{C}=\Omega _{er}=\frac{-d _{er}|E _{2,0}|}{h }$. After applying the rotating wave approximation, the hamiltonian of the system can be written as

3 $$\begin{aligned} H=\frac{\hbar }{2}\left( \begin{array}{ccc} 0&{}\Omega _{p}&{}0\\ \Omega _{p}&{}-2\Delta _{C}&{}\Omega _{C}\\ 0&{}\Omega _{C}&{}-2(\Delta _{C}+\delta _{P})\\ \end{array}\right) \end{aligned}$$

in which $\Delta _{C}=\delta _{er}$ and $\delta _{P}=\delta _{eg}$ are detunnings of coupling and probe lasers respectively. The evolution of the system can be described with Liouville-von Neumann equation:

4 $$\begin{aligned} {\dot{\rho }}=-\frac{i}{\hbar }[H,\rho ]+\mathcal {L}(\rho ) \end{aligned}$$

where the dissipation of the system is accounted for by the Lindblad operator

5 $$\begin{aligned} {\mathcal {L}(\rho )=\left( \begin{array}{ccc} \Gamma _{eg}\rho _{ee}&{}-\frac{1}{2}\Gamma _{eg}\rho _{ge}&{}-\frac{1}{2}\Gamma _{re}\rho _{gr}\\ -\frac{1}{2}\Gamma _{eg}\rho _{eg}&{}-\Gamma _{eg}\rho _{ee}+\Gamma _{re}\rho _{rr}&{}-\frac{1}{2}(\Gamma _{re}+\Gamma _{eg})\rho _{er}\\ -\frac{1}{2}\Gamma _{re}\rho _{rg}&{}-\frac{1}{2}(\Gamma _{re}+\Gamma _{eg})\rho _{re}&{}-\Gamma _{re}\rho _{rr}\\ \end{array}\right) } \end{aligned}$$

in which $\Gamma _{eg}$ and $\Gamma _{re}$ are natural decay rates from $|e\rangle$ to $|g\rangle$ and $|r\rangle$ to $|e\rangle$ respectively. In the experiment, we measured the transmission of probe laser which is related to the imaginary part of susceptibility of probe beam and is also proportional to the coherence $\rho _{eg}$ between ground and intermediate state. As the two laser beams are continuously applied to the atoms, the master Eq. (4) can be analytically solved to get solutions in steady state (i.e. ${\dot{\rho }}$ = 0) combining the condition of completeness $\sum \rho _{ii}$ = 1. In the case of a perturbative probe laser, It is sufficient to take only the first-order expansion in the probe Rabi frequency^26^.

6 $$\begin{aligned} {I m\rho _{ge}\propto \frac{4\delta ^{2}\Gamma _{eg}+\Gamma _{re}(|\Omega _{C}|^{2}+\Gamma _{eg}\Gamma _{re})}{||\Omega _{C}|^{2}+(\Gamma _{eg}-2i \delta _{P})(\Gamma _{re}-2i \delta )|^{2}}} \end{aligned}$$

where $\delta =\delta _{P}+\Delta _{C}$. For $6P_{3/2}$ state of cesium atom, $\Gamma _{eg}$ = 2$\pi \times$ 5.23 MHz^27^, and $\Gamma _{re}$= 2$\pi \times$ 3.97 kHz taking 40D state of cesium atoms as an example^21^. While for higher states, $\Gamma _{re}$ will become even less as the lifetimes of Rydberg states are proportional to $n ^{*3}$^1^.

## Results and discussions

The critical parameter of the experiment is Rabi frequency of coupling laser which determines the transmission of probe beam as we can see from Eq. (6).

7 $$\begin{aligned} {\Omega =\frac{\mu _{23}}{\hbar }\sqrt{\frac{2P}{\pi \omega ^{2}_{0}c\varepsilon _{0}}}} \end{aligned}$$

As shown above, the Rabi frequency can be divided into two parts, one of which is light part represented by power P and size of laser $\omega _{0}$, and the other one is atom part which is dipole matrix element $\mu _{23}$ between states $|e\rangle$ and $|r\rangle$. Since the transition dipole matrix element consists of radial and angular parts of which the angular momentum is related to the polarization of laser straightforward. We applied two quarter-wave plates with coatings of corresponding wavelengths to make different combinations of probe and coupling lasers as linear-linear ($\pi -\pi$), left-left circular ($\sigma ^{+}-\sigma ^{+}$), right-right circular ($\sigma ^{-}-\sigma ^{-}$), left-right circular ($\sigma ^{+}-\sigma ^{-}$) and right-left circular ($\sigma ^{-}-\sigma ^{+}$) polarizations, respectively. The power of probe and coupling lasers are varied in the range of 2 $\sim$ 8 $\upmu$W and 5 $\sim$ 60 mW independently.

**Figure 2:**
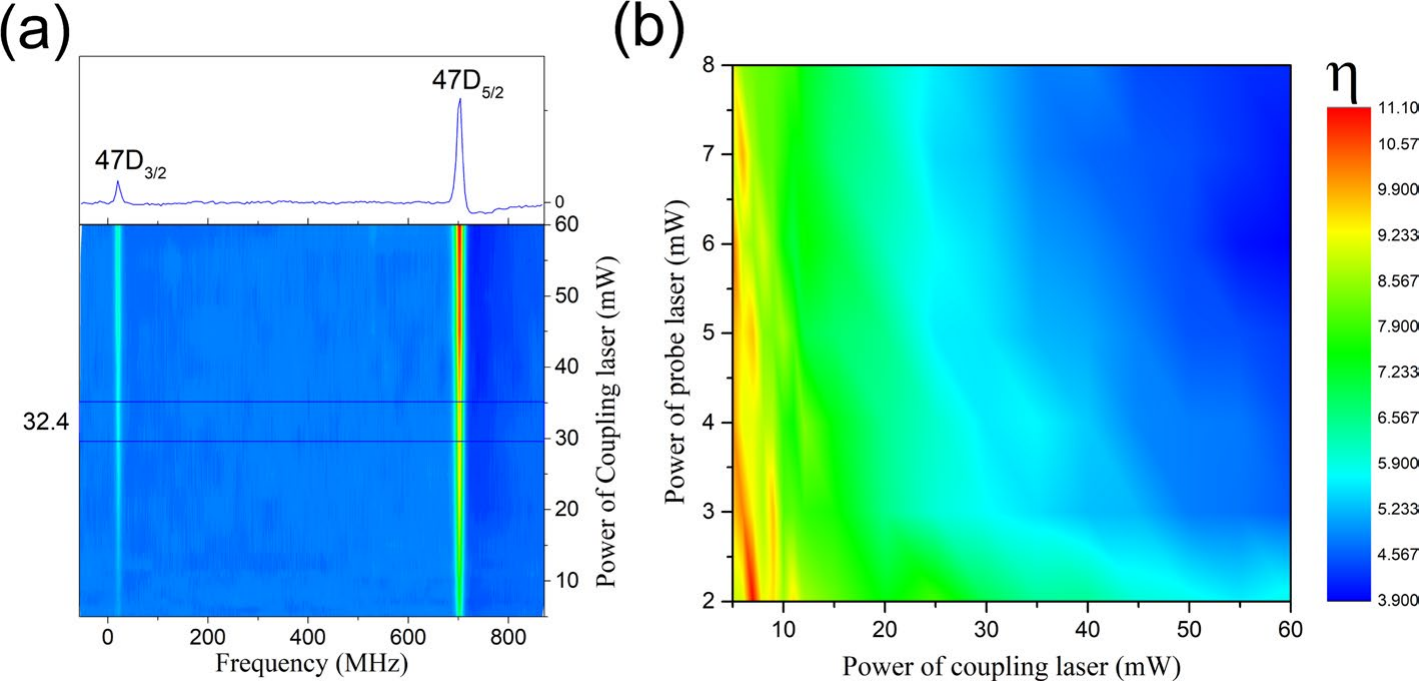
(**a**) Typical EIT spectrum of 47D$_{5/2,3/2}$ Rydberg states with polarizations of probe and coupling lasers as $\pi -\pi$ combination. (**b**) Dependence of transition strengths ratio $\eta$ between 47D$_{5/2}$ and 47D$_{3/2}$ states on power of probe and coupling lasers with polarization combination of $\pi -\pi$.

**Figure 3:**
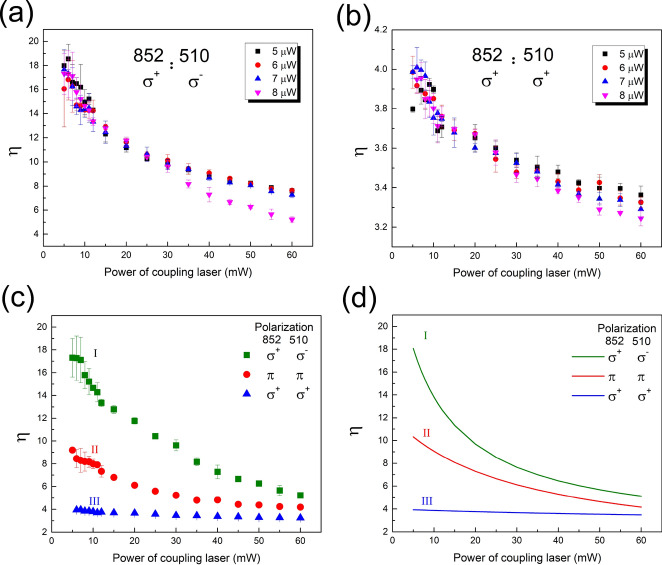
Dependence of transition strengths ratio $\eta$ of 47D$_{5/2}$ versus 47D$_{3/2}$ on power of 510 nm coupling laser with different combinations of probe and coupling lasers, where (**a**) for $\sigma ^{+}-\sigma ^{-}$ and (**b**) for $\sigma ^{+}-\sigma ^{+}$ under the conditions of power of probe laser ranging from 5 to 8 $\upmu$W. (**c**) and (**d**) are experimental data and theoretical simulation of dependence of $\eta$ on P$_{coupling}$ when P$_{probe}$ = 8 $\upmu$W with polarization combinations of probe and coupling laser as I:($\sigma ^{+}-\sigma ^{-}$), II:($\pi$-$\pi$) and III:($\sigma ^{+}-\sigma ^{+}$).

In the experiment, different Rydberg states can be accessed via tuning the frequency of 510 nm laser as is described in section “Experimental setup”. We first scanned the frequency of the green laser in the vicinity of 47D$_{5/2}$ and 47D$_{3/2}$ Rydberg states. Typical EIT spectroscopy of these two states is shown in Fig. 2a with power of probe laser 8 $\mu$W and coupling laser changed. It is clear to see that the signal of 47D$_{3/2}$ is much less than that of 47D$_{5/2}$ which illustrates the significant difference of transition strengths of these two Rydberg states. Here we empirically introduce a parameter $\eta$ = $\frac{S_{nD5/2}}{S_{nD3/2}}$, where S is intensity of the signal. Then the dependence of $\eta$ on the power of probe and coupling lasers ($\pi -\pi$ polarized) is shown in Fig. 2b which indicates that the rabi frequency of coupling laser has more influence than that of probe beam. Similar results are observed and shown in Fig. 3a and b. The combinations of polarizations of probe and coupling laser are $\sigma ^{+}-\sigma ^{-}$ and $\sigma ^{+}-\sigma ^{+}$ when the power of probe laser is changed from 5 to 8 $\upmu$W.

As mentioned above, the angular part of the transition dipole matrix element is driven and controlled by the polarization of excitation laser which will further affect the strength of allowed transition between states with certain angular quantum numbers. Then we investigate the behaviors of $\eta$ dependence on power of coupling laser under different combinations of polarization of probe and coupling lasers of three groups, which are I ($\sigma ^{+}-\sigma ^{-}$), II ($\pi -\pi$) and III ($\sigma ^{+}-\sigma ^{+}$). The transition path under excitations of $\sigma ^{-}-\sigma ^{+}$ (or $\sigma ^{-}-\sigma ^{-}$) is the mirror of that of $\sigma ^{+}-\sigma ^{-}$ (or $\sigma ^{+}-\sigma ^{+}$). So the data shown in Fig. 3c and d focus on three combinations of laser polarizations of $\sigma ^{+}-\sigma ^{-}$ and $\sigma ^{+}-\sigma ^{+}$ and the linear-linear one. The theoretical results are normalized to the maximum value of experiment data. Considering the circumstance of type I polarization, i.e. $\sigma ^{+}-\sigma ^{-}$, the polarizations of the probe and coupling laser are opposite. However, the two lasers are counter-propagated through the cell, then both of them drive the transitions which obey the rule of $\Delta m_{j}$ = 1. The laser we used in the experiment are continuous waves which means that the interaction time between light and atoms is sufficiently long compared to the lifetime of atomic states involved. So the effect of optical pumping is maximized. All of these analysis indicate that the transition 6S$_{1/2}$ $\rightarrow$ 6P$_{3/2}$ $\rightarrow$ 47D$_{3/2}$ or 47D$_{5/2}$ states preferred the path of 6S$_{1/2}$(F = 4, m$_{j}$ = -1/2) $\rightarrow$ 6P$_{3/2}$(F’ = 5, m$_{j}$ = 1/2) $\rightarrow$ 47D$_{3/2}$(m$_{j}$ = 3/2) for 47D$_{3/2}$ state or 6S$_{1/2}$(F = 4, m$_{j}$ = 1/2) $\rightarrow$ 6P$_{3/2}$(F’ = 5, m$_{j}$ = 3/2) $\rightarrow$ 47D$_{5/2}$(m$_{j}$ = 5/2) for 47D$_{5/2}$ state. The angular part of transition dipole matrix element of former transition is much less than that of the latter one, so the EIT signal of 47D$_{5/2}$ is much larger than that of 47D$_{3/2}$, that leads to larger $\eta$. For situation II, the transition rule become $\Delta m_{j}$= 0, the transitions for 47D$_{3/2}$ and 47D$_{5/2}$ states are 6S$_{1/2}$(F = 4, m$_{j}$=±1/2) $\rightarrow$ 6P$_{3/2}$(F’ = 5, m$_{j}$ = ± 1/2) $\rightarrow$ 47D$_{3/2}$ or $_{5/2}$(m$_{j}$=±1/2). The strengths difference between signals of D state splitting become less obvious due to the decreasing of transition dipole moment differences. For the last circumstances of III, the first transition from ground to intermediate state obey the rule of $\Delta m_{j}$ = 1, while the second one from intermediate to Rydberg state follows the rule of $\Delta m_{j}$ = − 1 since the green laser is count-propagated with respect to the IR one and the polarization is actually opposite. Under the influence of the maximized optical pumping effect, the preferred transitions for 47D$_{3/2}$ or 47D$_{5/2}$ states are 6S$_{1/2}$(F = 4, m$_{j}$ = 1/2) $\rightarrow$ 6P$_{3/2}$(F’ = 5, m$_{j}$ = 3/2) $\rightarrow$ 47D$_{3/2}$ or $_{5/2}$(m$_{j}$ = 1/2), the difference of transition dipole matrix elements for these two transitions is decrease further, so it’s the same tendency for $\eta$.

**Figure 4:**
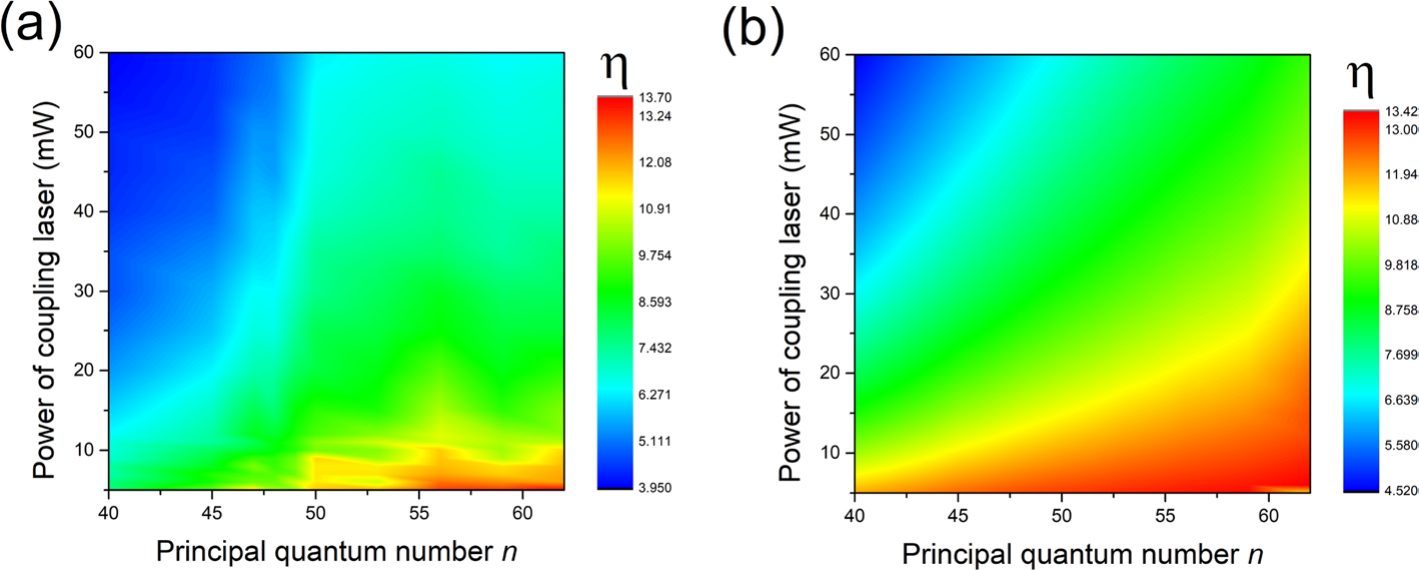
(**a**) Experimental results and (**b**) theoretical calculations of dependence of transition strength ratio $\eta$ of *n*D$_{5/2}$ versus *n*D$_{3/2}$ state on the power of coupling laser with principal quantum number *n* varying from 40 to 62. The polarizations of both lasers are linearly polarized.

At last we have measured the dependence of $\eta$ on power of coupling laser with principal quantum number *n* ranging from 40 to 62. Both of the probing and coupling lasers are linearly polarized in this case. Theoretical calculations and experimental results are shown in Fig. 4. The calculations show a nonlinear dependence of $\eta$ on *n* when the power of coupling laser is low, which indicate that the transition dipole matrix elements $\mu$ is proportional to $n ^{2}$, however it can be seen a saturation behavior of dependence of $\eta$ on *n* as in the regime of low power of coupling laser from the experimental results. We attribute this saturation to the strong interactions between Rydberg atoms which increase greatly as *n* rises. Since the effect of strong interactions between Rydberg atoms is excluded from the theoretical model, the saturation behavior of $\eta$ is not seen in the calculations.

## Conclusion

We have investigated the relationships between the transition strengths ratios $\eta$ and various experimental conditions including power of coupling laser and polarization combinations of probing and coupling lasers, respectively. The measured $\eta$ shows the nonlinear decreasing dependence on power of coupling laser with different polarizations of probing and coupling lasers as I ($\sigma ^{+}-\sigma ^{-}$), II ($\pi -\pi$) and III ($\sigma ^{+}-\sigma ^{+}$). Finally the dependence of $\eta$ on the principal quantum number *n* is also measured which shows an clear increasing of $\eta$ as *n* rises. The saturation of the experimental data indicates the influence of strong interactions between Rydberg atoms which will be investigated in the future work.

## Data Availability

The datasets used and/or analysed during the current study are available from the corresponding author on reasonable request.
